# Endosomal-Lysosomal Processing of Neurodegeneration-Associated Proteins in Astrocytes

**DOI:** 10.3390/ijms21145149

**Published:** 2020-07-21

**Authors:** Ching-On Wong

**Affiliations:** Department of Biological Sciences, Rutgers University, Newark, NJ 07102, USA; chingon.wong@rutgers.edu

**Keywords:** astrocytes, endosome, lysosome, neurodegenerative diseases, APP, amyloid beta, ApoE, tau, alpha synuclein, huntingtin

## Abstract

Most common neurodegenerative diseases (NDs) are characterized by deposition of protein aggregates that are resulted from misfolding, dysregulated trafficking, and compromised proteolytic degradation. These proteins exert cellular toxicity to a broad range of brain cells and are found in both neurons and glia. Extracellular monomeric and oligomeric ND-associated proteins are taken up by astrocytes, the most abundant glial cell in the brain. Internalization, intracellular trafficking, processing, and disposal of these proteins are executed by the endosomal-lysosomal system of astrocytes. Endosomal-lysosomal organelles thus mediate the cellular impact and metabolic fate of these toxic protein species. Given the indispensable role of astrocytes in brain metabolic homeostasis, the endosomal-lysosomal processing of these proteins plays a fundamental role in altering the trajectory of neurodegeneration. This review aims at summarizing the mounting evidence that has established the essential role of astrocytic endosomal-lysosomal organelles in the processing of amyloid precursor proteins, Apolipoprotein E (ApoE), tau, alpha synuclein, and huntingtin, which are associated with NDs such as Alzheimer’s, Parkinson’s, and Huntington diseases.

## 1. Introduction

The brain is an energy-demanding organ. Neurons require copious amount of ATP to repolarize membrane potential after neuronal firing. While this rapid demand of ATP is attained by neuronal mitochondria, the accompanied generation of reactive metabolites, such as oxidized fatty acids, pose a toxicity risk to neurons [1]. Also, storage and processing of energy precursor molecules require additional repertoire of organellar functions. To compartmentalize these metabolic functions within the brain, the tasks are delegated to the supporting cell astrocytes. Astrocytes provide lactate and glutamine to neurons to support neuronal activity [2]. Cardiovascular supply of glucose to brain cells is also regulated by astrocyte–endothelial cell and astrocyte–pericyte interaction [3]. As a constituent of the tripartite synapse, astrocytes buffer the concentrations of ions and neurotransmitters in the synaptic cleft, thereby modulating synaptic transmission [4,5]. As metabolic supporting cells, astrocytes are responsible for storing glycogen and lipids and possess the organellar machinery to process them into energy precursor molecules [6]. Indeed, astrocytes are essential in maintaining brain lipid homeostasis. Lipoproteins and cholesterol are synthesized and secreted by astrocytes. Fatty acid metabolism of astrocytes has recently been found to be critical in mitigating neuronal excitation-induced oxidative stress [7]. Astrocytes also exert modulatory effects on other brain cell types by releasing trophic factors, such as brain-derived neurotrophic factor (BDNF), and cytokines. Distinct secretome profiles are exhibited in differential activation states of astrocytes [8,9]. Mounting evidence has revealed the roles of these reactive astrocytes in aging and neurodegeneration (ND) [8,9]. In addition to their secretory role, astrocytes are essential for clearance and processing of proteins from the extracellular space. Astrocytes endocytose ND-associated proteins such as amyloid beta (Aβ), tau, alpha-synuclein (αSyn), and process them using the endolysosomal machinery. Astrocytic regulation of the metabolic fate of these ND-associated proteins is a major factor contributing to the disease pathogenesis and progression.

## 2. Endosomal-Lysosomal Organelles as A Metabolic Processing Hub

The primary function of the endolysosomal system is to execute metabolic tasks, which include the uptake, processing, appropriation, degradation, and disposal of molecules. The endolysosomal system consists of multiple vesicular compartments that undergo fusion and fission for the exchange and trafficking of the cargo molecules. Early endosomes are the first compartment where internalized molecules are contained and sorted. Early endosomes also provide a signaling platform where cognate ligands and receptors congregate. Sorting and recycling of molecules to other compartments, such as plasma membrane and Golgi network, occur at the recycling endosomes. Molecules destined for a degradative pathway are sorted into late endosomes, where the luminal pH gradually decreases with the assembly of vacuolar-type H^+^-ATPase complex at the endomembrane. At the same time, the late-endosomal vesicles are pulled along the microtubules by motor proteins, directing the movement from the cell periphery to the perinuclear region [10]. Calcium ions released from the endosomal-lysosomal organelles, hereinafter referred to as endolysosomes, trigger both motor protein-driven trafficking and SNARE-dependent vesicle fusion [11]. Fusion of late endosomes with autophagosomes, which contain organelles and high-molecular-weight proteins, brings the “garbage” together into the late-endosomal structure called amphisomes. Lysosomes, derived from the Golgi and packed with hydrolytic enzymes, fuse with amphisomes to form autolysosomes, where the proteolysis and lipolysis of luminal content occur. The resultant metabolic molecules, such as amino acids and fatty acids, are released into the cytosol or shuttled to other organelles, such as mitochondria and lipid droplets [12]. It appears that after the degradation, lysosomal enzymes are recycled and repackaged into regenerating lysosomes in a process termed lysosome reformation [13]. Also, it is evident that autolysosomes/lysosomes are capable of trafficking to the cell periphery and fusing with the plasma membrane [14]. This process serves the functions of plasma membrane repair and/or exocytosis of luminal molecules, such as lysosomal enzymes and processed proteins [15]. 

## 3. Astrocytes in Lysosomal Storage Diseases

Endolysosomal functions depend on a host of proteins residing in the vesicular lumen and membrane. Mutations to the genes encoding these endolysosomal proteins result in a group of congenital metabolic disorders called lysosomal storage diseases (LSDs). Many of the >60 LSDs display neuropathological phenotypes, highlighting the essential role of the endolysosomal system in brain cells. Endolysosomes are critical to the processing of metabolites in astrocytes, ensuring that reactive metabolites are sequestered from the neuron and converted into storage or energy precursors for neuronal use [7,16,17]. Endolysosomal dysfunction in LSDs thus impacts on the neuro-supportive role of astrocytes and exacerbates neurologic deficits. Mutations in *GBA1*, the gene encoding the lysosomal glucocerebrosidase, cause a common form of LSD called Gaucher disease. Carriers of *GBA1* mutations are also highly susceptible to developing Parkinson’s disease (PD) [18]. To examine the non-cell autonomous effect of *GBA1* mutations to neurodegeneration, a mouse model with astrocyte-specific knock-in of a disease-causing *GBA1* allele was created [19]. Primary astrocytes isolated from these mice show lysosomal storage accompanied by elevated secretion of pro-inflammatory cytokines [19], which might underlie the neuroinflammation observed in PD [20]. Loss-of-function mutations in *CLN3*, an endolysosomal transmembrane protein, result in a form of LSD called neuronal ceroid lipofuscinosis (NCL) [21]. Astrocytes isolated from a *CLN3* knockout mouse show diminished secretion of neuroprotective factors, and impaired calcium signaling and glutamate clearance [22]. Neurons co-cultured with these *CLN3*-deficient astrocytes display altered morphology and reduced survival [22]. Likewise, in another NCL model, in which the endolysosomal palmitoyl-protein thioesterase 1 (*PPT1*) gene is deleted, *PPT1*-deficient astrocytes also reduce viability of co-cultured neurons [23]. The non-cell autonomous effect on neuronal survival brought by astrocytic lysosomal storage is exemplified by an astrocyte-specific genetic model of LSD. Cre/Lox-mediated removal of the LSD gene sulfatase modifying factor 1 (*SUMF1*) in astrocytes induces neurodegeneration in vivo [24]. Astrocytic loss of *SUMF1* also results in distinct behavioral phenotypes compared to neuron-specific deletion, highlighting the astrocyte-specific roles contributing to the disease progression [24]. Interestingly, astrogliosis was observed in neuron-specific, but not astrocyte-specific, *SUMF1* knockout [24]. Although reactive astrocytes are commonly associated with neurodegeneration, their role in LSD-related neurodegeneration is unclear. Astrocyte activation is found to be attenuated upon loss of *CLN3* or *PPT1* [22,23]. Furthermore, preventing astrocyte activation by compound knockout of glial fibrillary acidic protein (GFAP) and Vimentin exacerbates disease progression in the *PPT1* model [25,26]. These findings suggest that endolysosomes are required for astrocyte activation that may play a protective role in neurodegeneration. 

Many neurodegenerative diseases (NDs) feature deposition of aggregate-prone proteins in the brain. Evidence has suggested that these proteins exert cellular toxicity on both neurons and glial cells. Uptake, processing, and release of these ND-associated proteins contribute to the intercellular propagation of cellular dysfunctions, leading to the collapse of the neuronal network [27]. It is becoming clear that glial cells, such as astrocytes, play an active role in the disease onset and progression brought by these ND proteins [28,29]. Astrocytic processing of these ND proteins not only influences their metabolic functions, but also the spreading of protein aggregation. The endolysosomal system is at a unique position where protein processing, metabolic regulation, and protein secretion intersect. Recent studies have unraveled the molecular underpinnings of how astrocytes utilize endolysosomes to process common ND proteins, including amyloid precursor protein, Apolipoprotein E (ApoE), tau, alpha synuclein, and huntingtin. This review aims at summarizing these recent findings. 

## 4. Amyloid Precursor Protein and ApoE Cross Paths in Endolysosomes

Elevated levels of amyloid beta (Aβ), extracellular deposition of Aβ plaques, and formation of intracellular neurofibrillary tangles in brain tissues are the most prominent pathological hallmarks of Alzheimer’s disease (AD). Aβ is derived from amyloid precursor protein (APP), a single-pass transmembrane protein enriched in neurons. APP protein can be found on the plasma membrane and endomembrane of trafficking vesicles [30], where it undergoes proteolytic processing by cleavage enzymes [31]. Cleavage by beta-secretase, also called beta-site APP cleaving enzyme 1 (BACE1), releases the extracellular domain of APP and results in a membrane-anchored C-terminal fragment (CTF) called APP-beta-CTF. Subsequent cleavage of beta-CTF by gamma-secretase, a complex that contains presenilin-1 (PSEN1), releases the transmembrane fragment and generates Aβ. Depending on where Aβ is generated and released, monomeric Aβ can be found in vesicular lumen or extracellular fluid such as cerebral spinal fluid and blood. The highly hydrophobic Aβ monomers can aggregate into soluble oligomers and insoluble amyloid fibrils, which are the main components of amyloid plaques. Due to the prevalence of Aβ accumulation in AD brains, it has long been proposed that Aβ generation is neurotoxic. 

### 4.1. Astrocytic Uptake of Extracellular Aβ and ApoE

**Figure 1:**
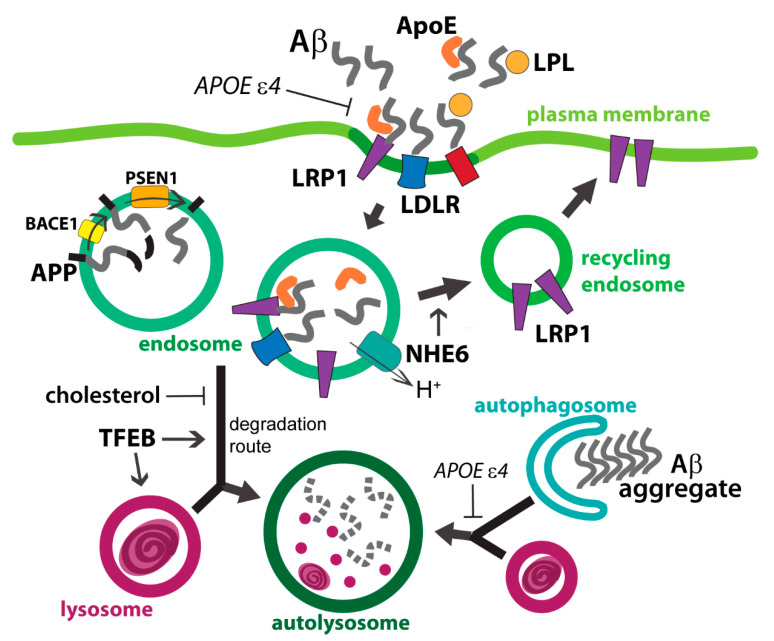
Uptake and endolysosomal processing of amyloid beta (Aβ) and Apolipoprotein E (ApoE) in astrocytes. Astrocytes internalize extracellular Aβ and ApoE via low-density lipoprotein receptor-related protein 1 (LRP1) and low-density lipoprotein receptor (LDLR) surface receptors. Dissociation of the ligand-receptor complexes takes place in the endosomal compartment, where free receptors are sorted and recycled back to plasma membrane. Proton leak via NHE6 is required for this recycling process. Aβ fragments are also generated by BACE1- and PSEN1-processing of APP in the endosomal compartment. TFEB promotes the lysosomal degradation of the Aβ-loaded vesicles. On the other hand, aggregates in the cytosol are recognized and contained in autophagosome, which subsequently fuses with lysosome.

Both neurons and astrocytes express APP, BACE1, and PSEN1, and are thus capable of generating Aβ fragments [32]. Neurons generate more Aβ than glial cells [33]. However, astrocytes in reactive states were found to produce more Aβ [34,35]. Astrocytes actively take up extracellular Aβ via clathrin-mediated endocytosis [36]. Interestingly, uptake of Aβ has been found to be mediated by proteins related to liproprotein uptake and metabolism (Figure 1). Surface receptors for lipoproteins have been found to facilitate Aβ endocytosis. Low-density lipoprotein receptor (LDLR) is necessary and sufficient for Aβ uptake and clearance by astrocytes [37]. Low-density lipoprotein receptor-related protein 1 (LRP1) was also found to be an Aβ receptor in astrocytes [38]. LRP1 expression is required for Aβ clearance in the APP/PS1 AD mouse model [38]. Apart from surface receptor, extracellular proteins also modulate Aβ endocytosis. Lipoprotein lipase (LPL) is responsible for extracellular hydrolysis of triglycerides in lipoprotein and is highly expressed in the brain. It was found that LPL promotes astrocytic uptake of Aβ, independent of ApoE [39]. 

Apolipoprotein E (ApoE), the major apolipoprotein of brain lipoprotein particles, is predominantly secreted by astrocytes and microglia. Extracellular ApoE can form a complex with Aβ and results in internalization via lipoprotein receptors (Figure 1) [40,41]. *APOE* alleles have long been known to be associated with AD. *APOE ε4* allele increases AD risk and the *ε2* allele is protective when compared with the most prevalent *ε3* allele. Consistent with the allelic difference in AD risk, ApoE isoforms of these three alleles were found to correlate with different Aβ clearance ability in cell and animal models. In a mouse model of Aβ-amyloidosis, clearance of Aβ from brain interstitial fluid was found to be highest in ApoE2 and lowest in ApoE4 [42]. Human astrocytes derived from induced pluripotent stem cells (iPSC) bearing the *APOE ε4* allele show reduced uptake of Aβ42 compared to *APOE ε3* astrocytes in isogenic background [43]. The ApoE isoform-dependent Aβ clearance is likely due to differential affinity between ApoE and the cognate lipoprotein receptors. Aβ complexes containing ApoE2 or ApoE3 were rapidly cleared from extracellular space via LRP1, whereas those with ApoE4 were internalized via very-low-density-lipoprotein receptor (VLDLR) at a slower rate [44]. Alternatively, direct internalization of Aβ by LRP1 in astrocytes can be hindered by ApoE-receptor binding, with ApoE4 found to exhibit the strongest hindrance [45]. 

### 4.2. Aβ and ApoE in Endosomal Compartment

How exactly ApoE impacts endolysosome trafficking machinery is not well defined. It was proposed that the acidic luminal environment of endolysosome promotes the unfolding of ApoE4 to form a molten globule state which is prone to aggregate with other proteins [46,47]. In neurons, higher affinity of ApoE4 for insulin receptors was found to prolong vesicular recycling of the receptors back to the plasma membrane [48]. Mobility of endolysosomes laden with different ApoE isoforms remains to be determined in astrocytes, however, it is plausible that ApoE4 differentially impedes endolysosome trafficking by a similar protein aggregation mechanism, especially in the context of ApoE-Aβ complex uptake. In addition, dysregulated ion homeostasis could synergize with the arrest of endosomal protein recycling. In ApoE4 astrocytes subjected to extracellular Aβ, the endosomal compartments are over-acidified as a result of epigenetic suppression of NHE6, which leaks protons from endosomes [49,50]. The abnormally low pH in the endosome traps LRP1 and prevents its recycling to the plasma membrane (Figure 1), therefore reducing the Aβ clearance ability [49]. Interestingly, endosomal acidification is accompanied by lysosomal alkalinization. Whereas NHE6 expression levels are lower in AD brains of *APOE* ε4 carriers, inhibition of histone deacetylases (HDAC) restores both NHE6 expression in ApoE4-knockin mouse astrocytes and their Aβ clearance ability [49]. Hence, endosomal ion homeostasis and/or epigenome of astrocytes could potentially be pharmacological targets for AD therapy. It should be noted, however, that promoting endosomal proton leak via NHE6 upregulation might not yield beneficial effects across all brain cell types. Neurons internalize ApoE via the Apoer2 receptor, which subsequently undergoes recycling at the endosomal compartment and gets redelivered to the cell surface. Xian et al. found that ApoE4 selectively causes endosomal retention of Apoer2 in neurons and prolongs Reelin signaling downstream of Apoer2 [46]. Pharmacological and genetic inhibition of NHE6 promote endosomal acidification and restore recycling of Apoer2 and Reelin signaling [46]. This is in contrast to astrocytic endosomes, where suppression of NHE6 arrests LRP1 cycling [49]. Since the astrocytic phenotype was caused by Aβ internalization, whereas the neuronal phenotype by ApoE4 internalization, the trafficking defect could be due to the differential effects of low pH to Aβ versus ApoE4. 

After endocytosis by astrocytes, Aβ molecules are contained in endosomes. Endosomal cargoes are sorted either to recycling endosome or to late endosome, which fuses with lysosome and undergoes enzymatic degradation (Figure 1). Endolysosomal trafficking is regulated by motor proteins which translocate vesicles along the cytoskeletal elements, such as microtubules and intermediate filaments (e.g., astroglia-specific glial acidic fibrillary protein (GFAP)) [51]. Reactive astrocytes, commonly found in multiple neurodegeneration models and pathological human brain tissue [8,9], exhibit faster endolysosome mobility via intermediate filaments [52]. Attenuated endolysosome mobility and vesicular atrial natriuretic peptide (ANP) secretion were found in astrocytes of 3xTg-AD mouse and astrocytes expressing mutant PSEN1 [53]. 

### 4.3. APP Fragments in Endolysosomes

Another source of intracellular Aβ in astrocytes is from cell-autonomous APP processing. BACE1, gamma-secretase, and Aβ fragments were found in astrocyte-derived exosomes isolated from human plasma [34]. Furthermore, levels of BACE1 and Aβ in these exosomes were significantly higher in the AD cohorts [34], supporting the notion that astrocytes can turn into major APP processing cell types in pathological conditions. It is likely that reactive astrocytes contribute to APP processing, since pro-inflammatory cytokines were found to upregulate APP and BACE1 expressions in astrocytes [54]. While APP can be cleaved at the plasma membrane where both BACE1 and gamma-secretase are localized to, mounting evidence has revealed that endolysosomes are the major site of APP processing (Figure 1) [55,56]. Endolysosomes serve as a congregating site for APP and BACE1 and provide an acidic environment that favors BACE1 activity [57,58]. Cleavage of APP in endolysosomes generates soluble APPβ and aggregate-prone Aβ fragments in the lumen, and APP C-terminal fragments (APP-CTFs) anchored to the limiting membrane. 

The APP fragments, together with other cargo proteins, in the endolysosomal compartments undertake two trafficking fates: release to the extracellular space via exocytosis or exosome secretion and shuttling to lysosomes for degradation. Whereas loss of Vps34 in neurons resulted in endolysosomal dysfunction and concomitant elevated secretion of exosomes bearing APP-CTF [59], the molecular pathway that generates Aβ-containing exosomes in astrocytes is yet to be determined [34]. Exocytosis of APP fragments generated at the endolysosomal compartments could either be a constitutive process or a consequence of intracellular accumulation due to impaired lysosomal degradation. Cholesterol sequestration by endolysosomes corresponds to decreased lysosomal clearance of APP fragments [60]. Lysosomal dysfunction is often linked with diminished autophagic flux, which is manifested in astrocytes expressing ApoE4 and correlates with accumulation of Aβ plaque [61], potentially exacerbating neuronal pathology. Indeed, incomplete lysosomal digestion of Aβ by astrocyte can promote non-cell autonomous apoptosis of neurons [62]. 

A number of factors can promote lysosomal degradation of APP fragments. Lipoprotein lipase and LDLR facilitate both endocytosis and lysosomal degradation of extracellular Aβ [37,39]. Promoting lysosome biogenesis in astrocytes effectively enhances lysosomal degradation of Aβ fragments in cell and animal models. Ectopic expression of Transcription Factor EB (TFEB), the regulator of lysosomal genes, in primary astrocytes or mouse model of AD enhances lysosomal degradation of Aβ and reduces amyloid plaque load [63]. Upregulating lysosome biogenesis in astrocytes by aspirin via the peroxisome proliferator-activated receptor alpha (PPAR)-TFEB pathway was also found to enhance lysosomal degradation of Aβ42 and clearance of Aβ plaque in a mouse AD model [64]. Together with the piling evidence for the beneficial effects of TFEB upregulation found in neurons, promoting TFEB activity and lysosome biogenesis appears to be a general strategy to mitigate AD hallmarks in animal models [65,66]. Considerations should be taken, however, that TFEB activity can amplify the p53 apoptotic pathway [67]. The aggregate clearance offered by TFEB may be rendered futile due to DNA damage, which is abundant in neurons during the onset phase of neurodegeneration [68]. 

## 5. The Tangle between Tau and Endolysosomes

Accumulation of protein aggregate species called neurofibrillary tangles (NFTs) is a hallmark in a wide array of neurodegenerative diseases that are collectively termed tauopathies [69]. NFTs consist of misfolded tau proteins, whose native function is to stabilize and promote microtubule assembly [69]. The intrinsic disordered domain of tau is sensitive to hyperphosphorylation which promotes the cytoplasmic formation of insoluble filamentous NFTs [69,70]. NFTs, along with hyperphosphorylated tau species, serve as a seeding platform for fibril elongation. Although NFTs are mainly found inside neurons, soluble and high-molecular-weight tau species are detected in interstitial fluid and cerebral spinal fluid of dementia patients and in media of cultured neurons [71,72,73]. Microglia-derived exosomes also contain tau, which promote intercellular tau propagation in animal models [74]. 

### 5.1. Tauopathy in Astrocytes

Tau is also endogenously expressed in astrocytes, albeit at lower levels compared to neurons [75]. Perinuclear tau deposits in astrocytes were found in brains of patients with Alzheimer’s disease (AD) and other neurodegenerative disorders [75]. It remains to be defined whether these tau inclusions come from endogenous expression or uptake from extracellular space. NFT formation in neuronal axon is known to cause vesicular and organellar trafficking defects [76,77]. In astrocyte, however, cellular impact of these tau inclusions is yet to be determined. It was found that presence of astrocytes with hyperphosphorylated tau and abnormal tau conformation correlates with altered phospho-proteome in brain cells [78]. Examinations of the thorn-shaped astrocytes in the human brains of aging-related tau astrogliopathy with no neuronal tau pathology suggested that tau seeding can occur in astrocytes [78]. In the P301S tau mouse model, which develops NFT hallmark along with neuronal loss, astrocytic markers such as GFAP and S100 calcium-binding protein B (S100β) are elevated, indicating astrogliosis [79]. Co-culture experiments demonstrated that expression of neuronal pro-survival factor thrombospondin 1 is diminished in P301S-tau astrocytes, resulting in reduction in synaptic markers in co-cultured neurons [79]. Hence, tauopathy in astrocytes may impact their neuro-supportive function and exert non-cell autonomous toxicity. 

### 5.2. Astrocytic Uptake and Processing of Tau

**Figure 2:**
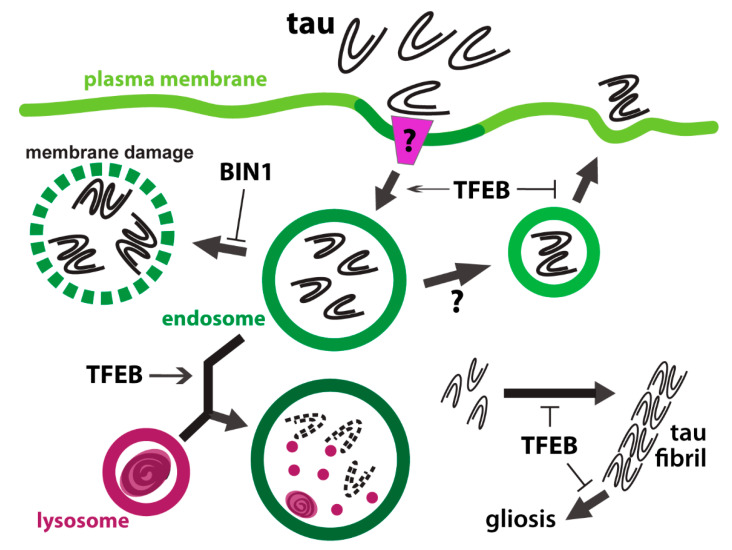
Endolysosomal processing of tau in astrocytes. Extracellular tau monomers and aggregates are endocytosed by astrocytes. Loss of BIN1, an Alzheimer’s disease-associated protein, promotes internalization and accumulation of tau aggregates in endosomes, and causes endomembrane damage. TFEB mediates multiple aspects of astrocytic clearance of tau. Uptake and lysosomal degradation of extracellular tau are enhanced by TFEB. TFEB activity is also required for limiting tau fibril formation and cell-to-cell transmission of tau.

Like endocytosis of Aβ, astrocytes are capable of internalizing extracellular tau (Figure 2). Primary rat astrocytes readily take up monomeric tau added to the culture medium [80]. Although heparan sulfate proteoglycans have been found to mediate the endocytosis of tau in neurons [81,82], it is not required for the astrocytic uptake of tau [80]. A yet-to-be-defined endocytic receptor in astrocyte might be responsible for tau uptake, similar to the CX3CR1-mediated tau uptake by microglia [83]. BIN1, the mutation of which is associated with AD [84], was found to regulate endocytic flux of tau aggregates in neurons and astrocytes [85]. Loss of BIN1 results in enhanced endocytosis of tau aggregates, which in turn damage endolysosomal membrane and trigger intracellular tau seeding (Figure 2) [85]. Neuronal suppression of BIN1 correlates with intercellular propagation of tau aggregates [85]. These findings suggest that endolysosomal processing of extracellular tau is required for intercellular spreading of tau pathology. It is not clear whether the internalized tau is transferred to other cells via recycling endosomes, multivesicular body-mediated exosomes, or transcytosis. However, enhancing lysosomal degradation appears to mitigate tau spreading. Overexpressing TFEB in primary mouse astrocytes enhances lysosomal biogenesis and concomitant uptake and lysosomal trafficking of tau fibrils (Figure 2) [86]. Using an adenovirus vector expressing TFEB under the GFAP astroglial promoter, two tauopathy mouse models were tested for the effect of astrocytic TFEB upregulation [86]. While astroglial TFEB overexpression did not alter pathological hallmarks in the aggressive rTg4510 model, levels of tau phosphorylation and inclusions, and gliosis, were all reduced in the slower progressing PS19 model [86]. Furthermore, tau spreading from the ipsilateral to the contralateral hippocampus was also attenuated upon astrocytic TFEB overexpression [86]. A concomitant increase in phosphorylated tau localized to the lysosomes of TFEB-overexpressing astrocytes suggests that astrocytes limit tau spreading by engulfing extracellular tau [86]. Together, these findings demonstrate that endolysosomes in astrocytes serve as a processing hub of extracellular tau and mitigate propagation of neuronal tauopathies. 

## 6. Alpha Synuclein

Alpha synucleinopathies are a host of neurodegenerative diseases characterized by cytoplasmic inclusions of alpha synuclein (αSyn) in the nervous system. Parkinson’s disease (PD) and Lewy body dementia are among the disease category. αSyn is encoded by the *SNCA* gene, which is abundantly expressed in neurons. The native function of αSyn is not entirely defined. By virtue of its interactions with membrane lipids and proteins, it has been found to modulate vesicular trafficking and synaptic vesicle release [87]. Similar to tau, the intrinsically disordered nature of αSyn makes it susceptible to oligomerization and fibril formation. *SNCA* mutations associated with PD result in αSyn variants with different kinetics of fibril formation [88]. Although intra-neuronal αSyn inclusions are the major disease hallmark, secretion of αSyn proteins to the extracellular space have been reported. αSyn is readily detected in the interstitial fluid of transgenic mice and human patients suffering from traumatic brain injury [89]. Multimeric αSyn complexes are detected in both culture media of primary neurons and interstitial fluid of mouse brains [90]. Interestingly, neuronal excitation seems to promote the secretion of these αSyn species both in vitro and in vivo [90]. It is not clear whether the high-molecular-weight complexes are formed prior or after release by neurons. Nevertheless, neuronal secretion of αSyn is regarded as an important mechanism for intercellular propagation of alpha synucleinopathy [91]. 

### 6.1. Astrocytic Uptake of αSyn

**Figure 3:**
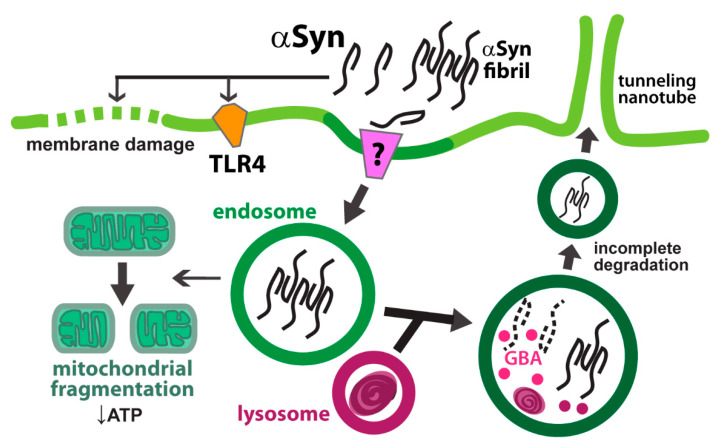
Endolysosomal trafficking and cellular impact of αSyn in astrocytes. Extracellular αSyn causes plasma membrane damage and activates cytokine production via toll-like receptor 4 (TLR4) in astrocytes. Accumulation of internalized αSyn fibrils in the endosomal compartments results in fragmentation of mitochondria and reduced ATP production. Lysosomal enzyme GBA is required for the degradation of αSyn. Incomplete degradation augments fibril formation and promotes cell-to-cell transmission via tunneling nanotubes, which connect to other astrocytes and neurons.

Primary astrocytes isolated from human brains are capable of taking up extracellular αSyn [92]. Prolonged αSyn treatment resulted in increased oxygen consumption and membrane damage in both primary neurons and astrocytes (Figure 3) [92]. It is still not clear what receptors mediate the internalization of extracellular αSyn. In contrast to microglial uptake [93], TLR4 has been found to be dispensable for the astrocytic uptake of αSyn [94]. However, astrocytic TLR4 is required for inflammatory signaling activation by αSyn [94]. Reactive oxygen species and inflammatory cytokines, such as IL-6 and TNF-α, were upregulated and released by astrocytes upon treatment with C-terminally truncated αSyn [94]. The heightened inflammatory signaling could be due to trafficking arrest of the endosomal compartments, where TLR4 signaling occurs [95,96]. Impact of internalized high-molecular-weight αSyn on other organelles has also been characterized. Astrocytes derived from mouse embryonic cortical stem cells readily take up extracellular oligomeric αSyn, and shuttle them to the endolysosomal compartments [97]. While the amount of internalized αSyn decreased initially, large amounts of undegraded αSyn-positive inclusion bodies persisted even after 12 days [97]. This indicates that lysosome cannot fully degrade the internalized αSyn fibrils (Figure 3). Concomitantly, mitochondrial fragmentation and reduced ATP level were observed in αSyn-treated astrocytes [97]. Given that astrocytes support neurons by providing metabolic precursors [6], defective bioenergetics in astrocytes resulted from αSyn inclusions likely exacerbates neuronal pathogenesis. 

### 6.2. Endolysosomal Processing of αSyn in Astrocytes

Fragmented mitochondria and impaired respiration also occur in astrocytes lacking glucocerebrosidase (GBA) [98]. Mutations in the *GBA1* gene result in Gaucher disease and increased susceptibility to Parkinson’s disease [99,100]. GBA is a membrane-associated enzyme localized to lysosome, where it removes the glucose moieties of membrane lipids such as glucosylceramide and glucosylsphingosine [18]. Loss of GBA function causes lysosomal storage and impaired autophagy in astrocytes [98]. While astrocytes are able to perform lysosomal degradation of internalized αSyn, such function is dependent on GBA activity (Figure 3) [101]. Astrocytes derived from Gaucher disease patients’ iPSC endocytose αSyn released by co-cultured neurons [101]. However, the impaired lysosomal degradative function in the disease astrocytes leads to further intracellular accumulation of αSyn [101]. How a lipid modifying enzyme affects αSyn degradation is not well understood. Alteration to the αSyn–membrane lipid interaction has been proposed as a mechanism of aggregation [102]. In support of this model, accumulation of glucosylsphingosine due to loss of GBA function has been found to trigger αSyn oligomerization [103]. Oligomeric αSyn in the endolysosomal lumen might resist degradation by lysosomal hydroxylases or aggregate with the enzymes and other lysosomal proteins. Interestingly, antibodies specific to αSyn oligomers could facilitate the uptake and subsequent lysosomal degradation in astrocytes [104]. Yet, antibody treatment after αSyn internalization cannot reduce pre-existing αSyn accumulation [104]. 

Astrocytes appear to have a higher αSyn degradative capacity compared to neurons [105]. In a astrocyte-neuron co-culture system, the astrocyte-to-neuron transfer of αSyn was found to be significantly less efficient compared to the neuron-to-astrocyte transfer [105]. αSyn in recipient cells are shuttled to the lysosomal compartment, where they are more efficiently degraded in astrocytes [105]. However, as illustrated above, intracellular accumulation and diminished lysosomal degradation can still occur when excessive αSyn are taken up by the astrocytes. Do astrocytes ramp up intercellular transfer to mitigate intracellular αSyn accumulation? Recent studies have found that astrocytes form tunneling nanotubes (TNTs) after lysosomal degradative function is compromised by the internalized αSyn oligomers (Figure 3) [106,107]. The receiving astrocytes in turn deliver mitochondria to the αSyn-laden astrocytes [106], potentially as a way to mitigate mitochondrial dysfunctions arisen from αSyn accumulation. While this study found that Golgi-derived vesicles are required for the intercellular transfer [106], others have found that αSyn fibrils are packaged in lysosomal vesicles and transported through TNTs [107]. Nevertheless, the physiological relevance of these cell culture-based findings is yet to be determined. In addition, even though intercellular transfer might serve as a self-rescue response by the astrocytes, it might be detrimental to the overall synucleinopathy progression. The transferred αSyn fibrils are capable of seeding aggregation of soluble αSyn in the otherwise healthy recipient cells [107], thus exacerbating αSyn fibril spreading as well as neuroinflammation [108]. If the astrocytic transmission is a stress-out response to lysosomal dysfunction, it will be interesting to test if enhancing lysosome biogenesis could reduce the intercellular spreading of αSyn. Of note, enhancing TFEB activity has been shown to reduce αSyn accumulation in neurons and oligodendrocytes [109,110].

## 7. Huntingtin and polyQ-Huntingtin

Huntington’s disease (HD) is a genetically inherited neurodegenerative disorder. HD is characterized by motor and cognitive deficits as the most prominent symptoms. The disease-causing mutation is an abnormal expansion of the CAG repeats in the *HTT* gene, which codes for huntingtin (HTT). When the CAG repeats exceed 35, the resulting extension of the polyglutamine (polyQ) stretch of HTT renders the protein pathogenic. HTT is expressed broadly across diverse cell types and can be found from the early developmental stage to adulthood. HTT interacts with a wide spectrum of proteins. Over 300 interacting partners involved in diverse cellular functions have been identified [111]. The protein is mainly localized to the cytoplasm, where it functions in regulating vesicular trafficking, cytoskeleton dynamics, proteostasis, and metabolism. By scaffolding the dynein/dynactin-kinesin complex, it regulates both the retrograde and anterograde trafficking of vesicles [112]. Trafficking of synaptic vesicles, endosomes, lysosomes, and autophagosomes have been found to be regulated by HTT [112]. Apart from vesicular trafficking, HTT also facilitates autophagic cargo recognition and promotes autophagy [113]. Mutant HTT impairs mitophagy by interrupting the recognition of damaged mitochondria by autophagosome [114]. Relatively low levels of HTT are localized to the nucleus, where it modulates gene transcription. HTT binds to a wide array of transcriptional factors, activators, and repressors [112]. Nuclear accumulation of HTT aggregates and the attendant transcriptional landscape alteration is therefore a prominent phenotype resulted from polyQ mutations [112]. Notably, brain cells from HD mouse models show differential levels of nuclear HTT inclusions, with neurons exhibiting much higher levels compared to astrocytes [115].

### 7.1. Mutant Huntingtin Arrests Vesicular Trafficking in Astrocytes

Neurologic phenotypes of a HD mouse model are alleviated by selectively reducing astrocytic expression of mutant HTT, demonstrating that astrocytes are a pathological contributor to HD [116]. Astrocytes generated from HD patient-derived iPSC display multiple cellular phenotypes resembling the features found in primary cells from HD patients [117]. These HD astrocytes exhibit profound cytoplasmic vacuole accumulation, which increases over time in culture [117]. Wild-type HTT is required for scaffolding the dynein motor complex to regulate endolysosomal trafficking [112,118]. Cytoplasmic accumulation of mutant polyQ-HTT might thus interfere with the assembly of motor proteins and stall vesicle movement along microtubule. Motor-driven vesicular trafficking is critical for astrocytes to provide a neuro-supportive function. Neural trophic factors such as BDNF are secreted by astrocytes via the endocytic vesicles [119]. Glial cells isolated from HD mouse models show reduction in both GDNF expression and BDNF secretion [120]. Ectopic expression of a truncated form of polyQ-HTT in rat cortical astrocytes also results in diminished BDNF secretion, which undermines neurite development of cultured neurons [121]. It was found that mutant HTT associates with Rab3a and impairs its function by preventing GTP hydrolysis [122]. This results in diminished plasma membrane docking of secretory vesicles that are packed with BNDF and ATP [122]. Apart from directly interfering with the vesicular trafficking machinery, mutant HTT also diminishes exosome secretion by suppressing transcription of exosome-related genes in astrocytes [123]. Hence, by disrupting the vesicular trafficking machinery, mutant HTT curtails the overall secretory function of astrocyte and undermines the neuro-protective and neuro-supportive functions mediated by astrocyte-derived trophic factors.

### 7.2. Astrocytic Clearance of Mutant Huntingtin

How do astrocytes get rid of HTT aggregates? Mutant HTT, but not wild-type HTT, is recognized and degraded by autophagy [124]. Pharmacological blockade of autophagosome fusion with lysosome inhibits the degradation of mutant HTT [124], suggesting that mutant HTT is shuttled to the lysosomal compartment. Interestingly, mutant HTT in neurons is targeting the lysosomal compartment for secretion [125]. While astrocytes degrade mutant HTT more efficiently than neurons [126], it is not known whether astrocytes also secrete mutant HTT via the late endosomal/lysosomal pathway. How astrocytes process extracellular mutant HTT remains unclear. Secreted mutant HTT may be endocytosed by astrocytes and seed aggregation, as observed in cell models [127]. Nevertheless, it appears that autophagy can effectively clear cytoplasmic mutant HTT aggregates. In line with this, the autophagy inducer trehalose was found to reduce the accumulation of mutant HTT in primary glial cells [120].

## 8. Concluding Remarks

Astrocytes actively internalize ND-associated proteins. It is evident that subsequent endolysosomal processing of these proteins alters cellular functions of the astrocytes. Incomplete degradation leads to seeding and aggregation of endogenous proteins, causing a jam in the vesicular trafficking system. By virtue of the metabolic roles of endolysosomes, cell metabolism and energetics can also be compromised, thus failing the neuro-supportive function offered by astrocytes. Alterations to cytokine expression and secretion also appear to be resulted from the attendant endolysosomal dysfunction. Defining the mechanism of how astrocytes are activated by endolysosomal processing of ND proteins will help elucidate the role of inflammatory response in NDs. On the other hand, it appears that promoting autophagic protein degradation and lysosome biogenesis facilitate astrocytic clearance of the ND proteins. However, transcriptional enhancement, for instance by TFEB activation, of these proteolytic processes also upregulates lysosomal exocytosis [128]. Such exocytic outlet of the endolysosomal pathway might accelerate transcellular spreading of the undegraded toxic protein species. Lastly, differential cell type-specific endolysosomal processing, for instance NHE6-mediated endosomal sorting, of ND proteins between neurons and astrocytes reveals another layer of complexity to the understanding of the disease mechanism. While there is a growing body of evidence showing unique roles of the astrocytic endolysosomal system in ND, more studies using cell type-specific models will advance our mechanistic understanding of astrocytic endolysosomes and provide insight into developing disease-modifying therapeutic strategies that target endolysosomes.
